# Mapping a Quantitative Trait Locus (QTL) conferring pyrethroid resistance in the African malaria vector *Anopheles funestus*

**DOI:** 10.1186/1471-2164-8-34

**Published:** 2007-01-29

**Authors:** Charles S Wondji, John Morgan, Maureen Coetzee, Richard H Hunt, Keith Steen, William C Black, Janet Hemingway, Hilary Ranson

**Affiliations:** 1 Liverpool School of Tropical Medicine, Pembroke Place, Liverpool, L3 5QA, UK; 2 Vector Control Reference Unit, National Institute for Communicable Diseases, NHLS, 1 Modderfontein Road, Sandringham 2131, Johannesburg, South Africa; 3 Medical Entomology, Division of Virology & Communicable Diseases Surveillance, School of Pathology of the National Health Laboratory Service and the University of the Witwatersrand, Johannesburg, South Africa; 4 School of Animal, Plant & Environmental Sciences, University of the Witwatersrand, Johannesburg, South Africa; 5 Arthropod-Borne and Infectious Diseases Laboratory, Department of Microbiology, Immunology and Pathology, Colorado State University, Fort Collins, Colorado 80523, US

## Abstract

**Background:**

Pyrethroid resistance in *Anopheles funestus *populations has led to an increase in malaria transmission in southern Africa. Resistance has been attributed to elevated activities of cytochrome P450s but the molecular basis underlying this metabolic resistance is unknown. Microsatellite and SNP markers were used to construct a linkage map and to detect a quantitative trait locus (QTL) associated with pyrethroid resistance in the FUMOZ-R strain of *An. funestus *from Mozambique.

**Results:**

By genotyping 349 F_2 _individuals from 11 independent families, a single major QTL, *rp1*, at the telomeric end of chromosome 2R was identified. The *rp1 *QTL appears to present a major effect since it accounts for more than 60% of the variance in susceptibility to permethrin. This QTL has a strong additive genetic effect with respect to susceptibility. Candidate genes associated with pyrethroid resistance in other species were physically mapped to *An. funestus *polytene chromosomes. This showed that *rp1 *is genetically linked to a cluster of CYP6 cytochrome P450 genes located on division 9 of chromosome 2R and confirmed earlier reports that pyrethroid resistance in this strain is not associated with target site mutations (*knockdown resistance*).

**Conclusion:**

We hypothesize that one or more of these CYP6 P450s clustered on chromosome 2R confers pyrethroid resistance in the FUMOZ-R strain of *An. funestus*.

## Background

*Anopheles funestus *is widely distributed in Africa from south of the Sahara desert to northern South Africa [1]. The highly anthropophilic and endophilic behaviors of this vector make it an efficient vector of malaria, and in many places, parasite infection rates of *An. funestus *exceeds those of *An. gambiae*. In South Africa, indoor house spraying with the insecticide DDT had successfully eradicated malaria transmission by *An. funestus*. However, after DDT was replaced by pyrethroids in 1996, resistance to the latter insecticides developed rapidly, and there was a dramatic increase in malaria transmission in Kwazulu/Natal Province, accompanied by the finding of *An. funestus *resting inside insecticide sprayed houses [2]. Subsequent surveys of southern Mozambican *An. funestus *also showed high levels of resistance to a variety of pyrethroids (permethrin, deltamethrin and lambda-cyhalothrin) and the carbamate propuxur [3], suggesting that the pyrethroid resistant *An. funestus *in South Africa had arrived from neighbouring Mozambique where *An. funestus *has remained abundant [4] and transmits malaria perennially [5].

The two major causes of insecticide resistance are alterations in the target sites and increases in the rate of insecticide metabolism. Three enzyme families, the cytochrome P450s, the esterases and glutathione S-transferases (GSTs), are primarily responsible for metabolic resistance [6]. Both field populations and a laboratory-selected colony of *An. funestus *from Mozambique show elevated levels of cytochrome P450s in pyrethroid resistance populations [3]. Cytochrome P450-based pyrethroid resistance also occurs in East and West African *An. gambiae *[7,8] although in West Africa the P450 mechanism is at a much lower frequency than the altered target site [knockdown resistance (kdr)-type] resistance mechanism [7]. The appearance of a P450-based pyrethroid resistance in southern African *An. funestus *had an immediate and significant effect on malaria transmission in South Africa prompting a switch back to DDT in South Africa and the introduction of the carbamate, bendiocarb in Mozambique [2,3]. However, resistance to these alternative insecticides has already developed in *An. arabiensis *in South Africa [9] and hence effective resistance management strategies are imperative in this region. Identification and characterization of the specific P450 genes involved in pyrethroid metabolism in resistant insects is an important step towards this goal.

The cytochrome P450 enzyme family is very extensive in insects [10,11]. Only a small subset of these enzymes is likely to be involved in insecticide metabolism. Partial sequences of approximately thirty P450 genes from three families (CYP 4. 6 and 9) have already been identified in *An. funestus *[12], and here we report the physical mapping of a subset of these genes to the *An. funestus *polytene chromosomes. We also describe the first application of quantitative trait locus (QTL) mapping in this malaria vector. QTL mapping techniques have been successfully used in other mosquito vectors to examine complex phenotypes including vectorial competence [13,14] and insecticide resistance [15,16]. The QTL approach has never been used in *An. funestus *mainly because of the difficulties associated with colonization of this species and the lack of available genetic markers. However, the recent establishment of two laboratory colonies of *An. funestus *[17] and the construction of a preliminary genetic map [18] have paved the way for the current study. In this paper we report the genetic and physical map position of a QTL associated with pyrethroid resistance in *An. funestus*.

## Results

### Linkage mapping

**Figure 1:**
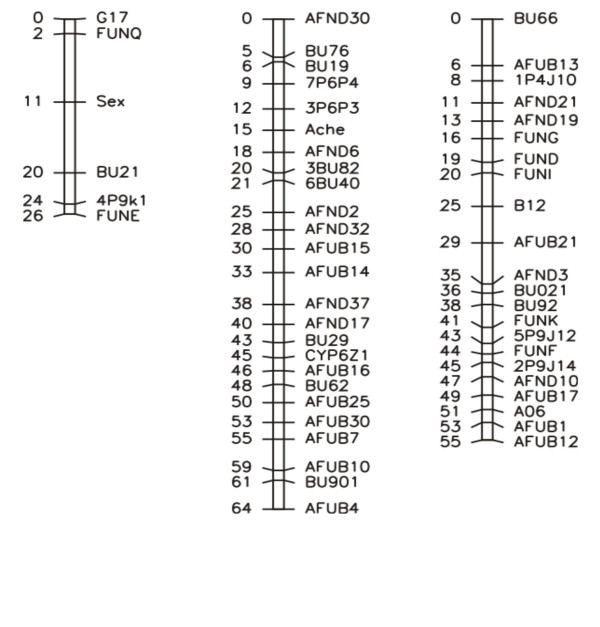
Genetic map of *An. funestus *using the combined data from the 11 families. A scale of genetic distance in centimorgans is shown.

Data from the eleven families were used to build a combined genetic linkage map by genotyping a total of 349 F_2 _individuals at 56 loci (Figure 1). Three linkage groups were resolved instead of four in the previous map [18], with each linkage group corresponding to one of the three chromosomes. The linkage map of the X chromosome with 6 markers was 26 cM in length compared to 44 cM for five markers previously. 25 markers were mapped to chromosome 2 for a genetic distance of 64 cM compared to 158 cM for 16 markers previously. One linkage group was resolved for chromosome 3 instead of two previously with 22 markers giving a genetic distance of 55 cM which is well below the total distance of 189 cM for 18 markers observed in the first map. The average resolution for each chromosome is 4.3, 2.5 and 2.5 cM respectively for chromosome X, 2 and 3. The total length of the current map is 145 cM with an average resolution of 2.7 cM/marker.

### QTL mapping

#### Genotype-phenotype associations

We used χ^2 ^goodness-of fit tests to identify loci statistically (*P *< 0.05) associated with pyrethroid resistance in each family, the null hypothesis being that resistance or susceptibility to pyrethroids is equal in each genotype class. Significant loci are indicated in Table 1.

**Figure 2:**
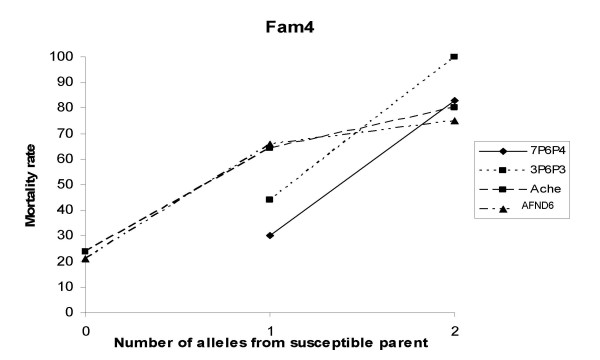
Plot of mortality rate as a function of alleles inherited from the susceptible parent in family 4 for significant markers on chromosome 2.

For family 4, we found significant associations between four loci and resistance to pyrethroids. Two of these loci (7P6P4 and 3P6P3) were semi-informative (one of the P_1 _parent being heterozygote while the other is homozygote for the locus) for this family and the others (*AChE *and AFND6) were fully informative. For 7P6P4, 83% of F_2 _individuals with an "aa" genotype (genotype of the parental susceptible female) and 30% of F_2 _heterozygous "ab" died after 24 h exposure to permethrin. These values are significantly different and also oriented in the anticipated direction. This trend is repeated for marker 3P6P3 where 100% of homozygous "aa" and 44% of heterozygous "ab" F_2 _individuals died after exposure to permethrin. For locus *AChE*, mortality rates of 80% for F_2 _"aa" homozygous individuals, 64% for heterozygous "ab" and 23% for homozygous "bb" (genotype of the parental resistant male) were obtained. A similar pattern is seen for locus AFND6 (Figure 2).

For family 11, statistically significant associations were found between genotypes at three loci (7P6P4, 3P6P3 and AFND6) and the resistance trait. These loci showed a similar pattern of association with pyrethroid resistance as in family 4. A high mortality rate was observed in "aa" homozygous F_2 _individuals (100, 60 and 100% respectively for 7P6P4, 3P6P3 and AFND6) while mortality was low in "bb" individuals (19 and 39%, respectively for 7P6P4 and AF6).

For family 6, only two markers (3BU82 and 6BU40) were statistically associated with pyrethroid resistance. Again, the mortality rate was higher in F_2 _"aa" homozygotes (65 and 67% respectively for 3BU82 and 6BU40) than in F_2 _"bb" homozygotes (25 and 35% respectively for 3BU82 and 6BU40).

**Figure 3:**
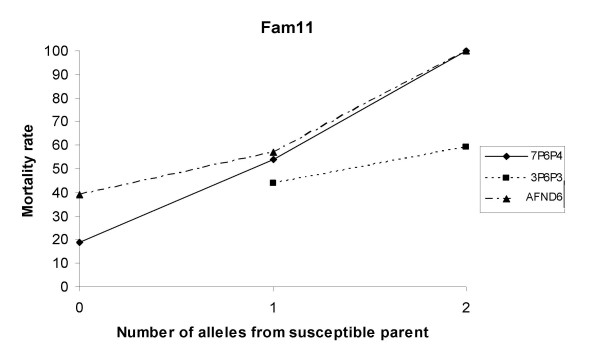
Plot of mortality rate as a function of alleles inherited from the susceptible parent in family 11 for significant markers on chromosome 2.

This analysis indicates some important factors linked to the inheritance of pyrethroid resistance trait. All four loci associated with the resistance trait are located on chromosome 2R (between divisions 9 and 12) suggesting that a locus or loci that affect resistance to pyrethroids is located within this region of the genome. Furthermore, plotting resistance against genotypes (Figures 2 and 3) also suggests that alleles at these loci are additive in their effect on resistance.

We next used interval mapping (IM), composite-interval mapping and multiple-interval mapping to predict the location of this QTL. Three families with a sample size of more than 40 F_2 _progeny (families 4, 6 and 11) were independently analyzed for the presence of QTL associated with pyrethroid resistance.

**Figure 4:**
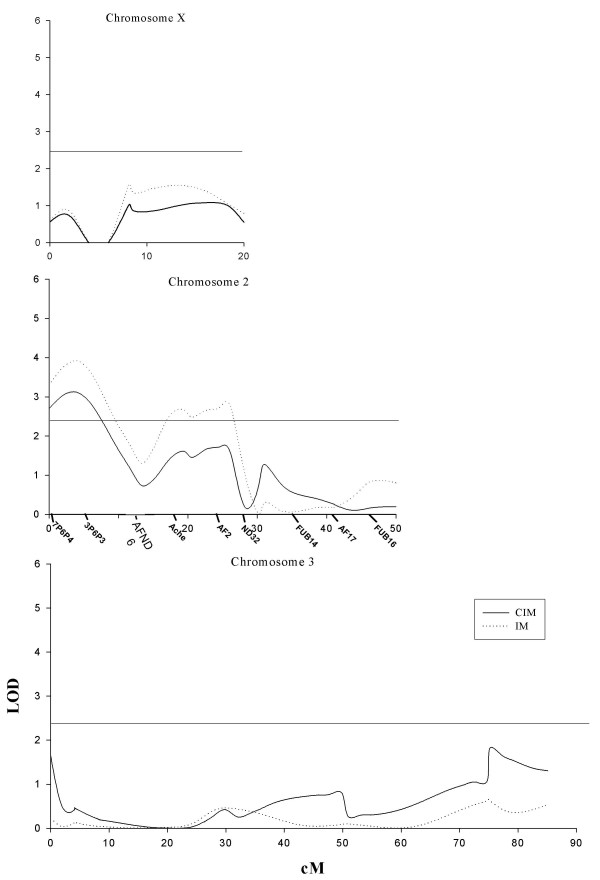
Plot of LOD scores associated with pyrethroid resistance on chromosomes X, 2 and 3 in family 4. Solid lines represent LOD estimated by composite-interval mapping and dashed lines represent LOD estimated by interval mapping. The straight line along the top of each graph represents the threshold value for LOD. Names of markers are listed on chromosome 2 to orient QTL position.

For family 4, a QTL was detected at the end of chromosome 2R with both IM and CIM (Figure 4) between markers 7P6P4 and 3P6P3 already found to be associated with pyrethroid resistance by χ^2 ^goodness-of fit tests. The LOD scores associated with this QTL are 3.3 and 4 by IM and CIM respectively. No QTL were detected on chromosome X and chromosome 3. Multiple-interval mapping confirmed the presence of this QTL (Table 2) and estimated that its genetic variance (σ_g_^2^) accounted for 27.9% of the phenotypic variance (σ_p_^2^) for pyrethroid resistance (27.9% resulting from 31.1% for additive effect and -3.2% for dominance effect). Most of the genetic variance (σ_g_^2^) was attributable to additive effects (31.1%, Table 2). Two additional QTLs were detected in this family after refining the MIM model by searching and testing for new QTLs. These were located at 20 cM on chromosome X between markers G17 and FUNQ and at position 75.2 cM on chromosome 3 between markers ND10 and FUB1 (Table 2). These QTL were not detected by any other analysis method, nor were they found in other single family analysis.

**Figure 5:**
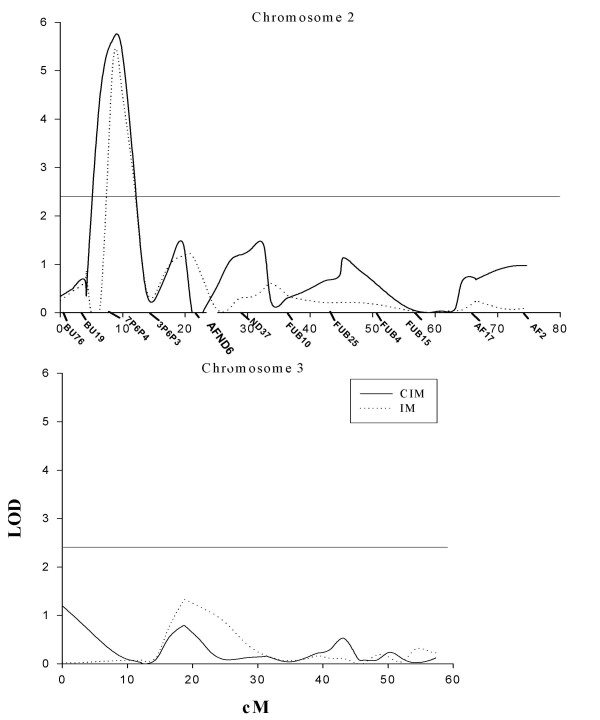
Plot of LOD scores associated with pyrethroid resistance on chromosomes 2 and 3 in family11. Solid lines represent LOD estimated by interval mapping and dashed lines represent LOD estimated by composite-interval mapping. The straight line along the top of each graph represents the threshold value for LOD. Names of markers are listed only on chromosome 2 to orient the unique QTL position.

For family 11, using IM and CIM, we detected a QTL on chromosome 2R between markers 7P6P4 and 3P6P3, at a similar position to that in family 4. This QTL was located at 7.3 cM with 7P6P4 as the nearest marker. The LOD scores for this QTL were 5 and 5.8 with IM and CIM respectively (Figure 5). With MIM, only one significant QTL was detected which, in this family, accounted for 63.4% of phenotypic variance (σ_p_^2^) (Table 2). Additive effects accounted for the majority of the genetic variance. We have named this QTL *rp1 *for resistance to permethrin 1. No QTL was detected on chromosome 3 and no genetic linkage was constructed for chromosome X in this family due to a lack of informative markers on this chromosome.

In families 6 and 10 (data not shown in Table 2 for family 10 because of the low sample size), the markers 7P6P4 and 3P6P3 were not informative and only low LOD scores were observed at markers at the end of 2R chromosome. However when using multiple-interval mapping, we detected the same QTL as that observed in families 4 and 11. The LOD score for this QTL in family 6 and 10 was 1.9 and 1.6 respectively with composite-interval mapping, which is considerably smaller than that observed for families 4 and 11. This is probably due to the small sample size (family 10) and the lack of informative markers in this region of the genome. In families 4 and 11, as in family 6 (Table 2), additive effects accounted for the majority of the genetic variance.

### Physical mapping of *rp1 *QTL

**Figure 6:**
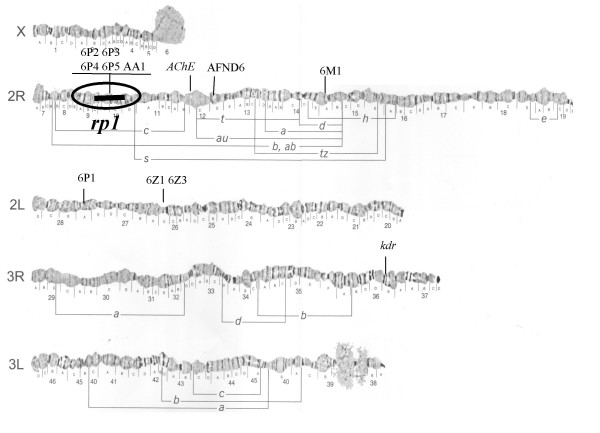
Cytogenetic map of *An. funestus *polytene chromosomes indicating *rp1 *QTL position and P450 genes and insecticide target site genes, physically mapped by *in situ *hybridisation during this study. This figure is adapted from [39].

The genes implicated in insecticide resistance in other species (cytochrome P450s, *kdr*, *AChE*) were physically mapped to the *An. funestus *polytene chromosomes in order to locate possible candidate resistance genes within the boundaries of the QTL. The location of these genes is shown in Figure 6 and the map position of these genes and of their orthologs in *An. gambiae *is shown in Table 3. There is a high level of synteny between *An. funestus *and *An. gambiae *in the location of these genes. With the exception of CYP6M1 and CYP6P1, all of the *An. funestus *probes hybridized to the expected chromosome arms according to the established synteny between the two species [19]. CYP6M1 in *An. funestus *is found on chromosome 2R instead of the predicted 2L and CYP6P1 was located on 2L arm and not 2R as expected.

Five CYP6 P450s physically mapped to chromosome arm 2R, within the region of *rp1 *QTL (Figure 6). These included CYP6P4 and CYP6P3 from which two SNP loci, (7P6P4 and 3P6P3) were identified. In agreement with the physical mapping data, we found that these SNPs were closely linked to *rp1 *in all families for which these markers were informative.

The *kdr *locus is located on chromosome 3R, division 36, and is not linked to pyrethroid resistance in the FUMOZ-R strain of *An. funestus*. Again this agrees with the chromosomal location determined for this gene in *A. gambiae *(3R in *An. funestus *is equivalent to 2L in *A. gambiae*). No amino acid substitutions were found in the amplified 1342 bp fragment of the voltage gated sodium channel gene in the FOMUZ-R strain compared to the field samples of *An. funestus *and the classical *kdr *leucine substitution at position 1014, associated with pyrethroid resistance in many insect species, was not found in *An. funestus*.

The fragment of the *AChE *gene sequenced encompasses the common *ace-1 *mutation site associated with insensitive *AChE *in *An. gambiae *[20]. We did not detect this mutation in the *An. funestus AChE*.

## Discussion

To detect QTL associated with resistance to pyrethroids in *An. funestus*, we constructed a linkage map of this species using combined data from 11 families generated from reciprocal crosses between a susceptible and permethrin resistant strain. The average resolution of 2.7 cM/marker is similar to the resolution of the published *An. gambiae *microsatellite map [21]. The present *An. funestus *genetic map uniformly covers the entire genome of *An. funestus *with a similar marker density for chromosomes 2 and 3. This genetic map represents a significant improvement from the previous published map of [18].

The *rp1 *QTL that affects *An. funestus *susceptibility to pyrethroids was consistently identified in both reciprocal crosses and in the majority of the families (7 out of 11), demonstrating the importance of this QTL. Generally the LOD scores associated with the *rp1 *QTL were well above the thresholds determined by permutation analysis using both interval mapping and composite-interval mapping. The *rp1 *QTL was most closely associated with markers 7P6P4 and 3P6P3 on chromosome 2R regardless of the analytical method employed (IM, CIM and MIM). Such correlation between different methods and consistency between different families represent a strong indication that the QTL identified in this study reflects the detection of a true locus involved in resistance to pyrethroids in *An. funestus*. The percentage of phenotypic variance explained by *rp1 *differed significantly between families. The different genetic backgrounds of these families could explain the differences observed. The variance explained by *rp1 *in family 11 (63.4%) could also represent a better reflection of the percentage of variance explained by this QTL than that observed in family 4 (27.9%), as the two closest markers to *rp1*, 7P6P4 and 3P6P3, were fully informative in family 11 but only semi informative in family 4, hence, only half of the genetic information at these loci was available in family 4. The *rp1 *QTL appears to present a major effect since it explains more than 60% of the variance for pyrethroid resistance. In general, our results suggest that resistance to pyrethroids is a quantitative trait under the control of at least one QTL.

The family sizes used in this study were generally low because it is difficult to colonize *An. funestus*. The low sample sizes may have prevented the identification of additional QTLs of minor effect. Small sample sizes are more adequate to detect QTL with large effect such as *rp1 *but have limited power to detect smaller QTLs [22-24]. This may explain why only one confirmed QTL has been identified in this study. A denser marker map might also have a greater power to detect minor QTL and to separate any linked genes located within *rp1*. The numbers of actual QTL have been underestimated in studies involving QTL of large effect [25].

In family 4, two additional QTLs other than *rp1 *were identified on chromosome 2 by multiple-interval mapping. However when the genotypes of markers linked with these QTLs were tested for association with pyrethroid resistance using the χ^2 ^goodness-of fit test, no association was found. Results from interval mapping and composite-interval mapping did not indicate the presence of QTL around the positions indicated by these MIM results. We believe that these additional QTLs are spurious and need to be confirmed in larger families with a greater number of genetic markers.

The region of the genome where the *rp1 *QTL was identified contains a cluster of cytochrome P450 genes belonging to the CYP6 family. Indeed, the two SNP markers (7P6P4 and 3P6P3) most closely linked to *rp1 *are located within two CYP6 genes. Elevated cytochrome P450 activity has been implicated as the major mechanism conferring pyrethroid resistance in *An. funestus *[3]. The molecular basis of cytochrome P450-mediated insecticide resistance may involve *cis *or *trans *regulation of the transcription of a P450 gene [26]. It is also possible, although uncommon, that resistance may be caused by a change in the P450 amino acid sequence increasing the affinity of a P450 for pyrethroids [27]. In *An. gambiae*, a QTL has been mapped in the vicinity of a P450 gene (CYP6Z1) shown to be over-expressed in a resistant strain [28]. To study the molecular mechanism of the pyrethroid resistance in the FUMOZ-R strain, and to see if additional minor pyrethroid resistance QTLs exist in the population, we are establishing advanced intercross lines and identifying additional genetic markers to better define the interval in which the *rp1 *QTL is mapped.

## Conclusion

The present study reports the first QTL mapping study in *An. funestus*, one of the most important malaria vectors. This study suggests that metabolic resistance mechanism is playing a significant role in pyrethroid resistance in *An. funestus *since we did not found any evidence either from direct sequencing or QTL mapping, that mutations in the target site are involved in pyrethroid resistance in *An. funestus*. The *rp1 *QTL identified here represents a first step toward a fine mapping of genes involved in this resistance trait and by combining this approach with functional characterization of P450 genes on chromosome 2R we will elucidate the molecular basis of pyrethroid resistance in this malaria vector.

## Methods

### Mosquito strains and bioassays

The two strains of *An. funestus *used in this study were colonized by the Vector Control Reference Unit of the National Institute for Communicable Diseases in South Africa [17]. The FUMOZ strain originated from southern Mozambique in 2000 and was initially a heterozygous permethrin resistant population. After selection of the parental strain with 0.1% lambda cyhalothrin [17], a highly resistant strain called FUMOZ-R was generated. The FANG colony which is completely susceptible to all pyrethroids was colonized from Calueque in southern Angola in 2002.

### Mosquito crosses

Reciprocal crosses using virgin FANG and FUMOZ-R females with males from the alternative strain were set-up. Blood fed, mated females were left to oviposit singly and the F1 were crossed to generate F_2 _progeny, producing iso-female lines. Bioassays were carried out on 1–3-day-old adults using the standard WHO bioassay procedure [29]. Mosquitoes were exposed to 0.75% permethrin for 1 hour and mortality was recorded 24 h post-exposure. Dead mosquitoes were considered as susceptible to permethrin and separated from the living progeny which were considered as resistant. Parental females (P_1_), F_1 _progeny as well as F_2 _progeny from each family were collected and kept in silica gel Eppendorf tubes for later DNA extraction and genotype determination.

### Genotyping of molecular markers

Progeny from 11 families were used in this study. Six of these originated from a FANG × FUMOZ-R cross and five from the reciprocal FUMOZ-R × FANG cross. In each case approximately equal numbers of surviving and dead progeny were genotyped (Table 4). DNA was extracted using the LIVAK method [30] and the microsatellite loci were genotyped as described previously [18]. A total of 72 microsatellite markers were scored in the P_1 _and F_1 _samples and the F2 progeny were scored for informative markers in each family using the Beckman CEQ8000 fragment analysis program.

### Single Nucleotide Polymorphisms (SNPs) genotyping

Fifteen SNP markers were previously identified in *An. funestus *[18]. These were scored in the P_1 _and F_1 _parents in each of the 11 families and informative SNPs were scored either by the heated ligation oligonucleotide assay (HOLA) [31] or by single-base pair extension reaction using terminator dyes and CEQ8000 software or using the pyrosequencing method, as described below. An additional 11 informative SNP markers were identified by direct sequencing of PCR products from cytochrome P450 genes or from genes that had been previously mapped to *An. funestus *polytene chromosomes [19].

### Pyrosequencing reactions

Three sequence-specific primers were designed for each marker (Table 5) using the software provided by Pyrosequencing AB. Forward and a biotinylated reverse primers (10 pmol) were added to a PCR reaction containing 1× HotStarTaq buffer, 0.2 mM dNTPs, 1.5 mM MgCl2, 1 U HotStarTaq (Qiagen) and 10 ng genomic DNA. Amplification was performed with the following conditions: 1 cycle at 95°C for 5 min; 50 cycles of 94°C for 20 s, 57°C for 30 s and elongation at 72°C for 20 s; followed by 1 cycle at 72°C for 5 min.

Single-stranded biotinylated PCR products were prepared for the pyrosequencing reaction using a Vacuum Prep Tool (Biotage AB). The biotinylated PCR products were immobilized onto Streptavidin Sepharose high performance beads. For a single sample, 3 μl of Streptavidin Sepharose™ HP (Amersham) were added to 37 μl binding buffer (10 mM Tris-HCl pH 7.6, 2 M NaCl, 1 mM EDTA, 0.1% Tween 20) and mixed with 25 μl PCR product and 15 μl water on a mechanical shaker for 5 min at room temperature in a 96-well plate. The beads containing the immobilised templates were captured onto the filter probes after applying the vacuum and then washed with 70% ethanol for 5 sec, denaturizing solution (0.2 M NaOH) for 5 sec and washing buffer (10 mM Tris-Acetate pH 7.6) for 5 sec. The vacuum was switched off and the beads were released into a PSQ 96 well plate containing 38.4 μl annealing buffer (20 mM Tris-Acetate, 2 mM MgAc2 pH 7.6) and 1.6 μl of the corresponding sequencing primer (10 μM). Annealing was achieved by heating the samples to 80°C for 3 min and then allowing them to cool to room temperature. Pyrosequencing reactions were performed according to the manufacturer's instructions using the PSQ 96 SNP Reagent Kit (Biotage AB) and the genotype was determined using SNP Software (Biotage AB).

### Linkage mapping

The JoinMap 2.0 package [32] was used to build a genetic linkage map for each individual family and for the combined genotype data from all families. Genotype data for each marker were tested for conformity to Mendelian ratio with a χ^2 ^goodness-of-fit analysis using the JMSLA procedure. Loci were separated into linkage groups using the JMGRP1 and JMSPL procedures with minimum and maximum LOD thresholds of 0.0 and 6.0 respectively and LOD increments of 0.1. The JMREC program estimated pairwise cM distances between all pairs of informative loci in each linkage group. The JMMAP program estimated the maximum likelihood map using the Kosambi distances. DrawMap 1.1 [33] software was then used to plot the genetic map.

### QTL mapping

Associations between genotypes at each locus and the resistance phenotype (dead or alive) were assessed using a contingency χ^2 ^analysis. The null hypothesis was that susceptibility to pyrethroid is equal in each genotype class. The marginal probabilities were the frequencies of each genotype at a locus and the mortality and survival rates after pyrethroid exposure. For loci with a significant χ^2 ^we analyzed the inheritance of the alleles at these loci. The *a priori *hypothesis was that higher mortality rate would occur among F_2 _individuals with one or both alleles inherited from the susceptible parent while lower mortality rate will be observed among individuals with alleles inherited from the resistant parent.

The JoinMap linkage map and the genotype/phenotype data were entered into Windows QTL Cartographer 2.5 [34]. Interval mapping [22], composite-interval mapping [24] and multiple-interval mapping [35] procedures were implemented for each family. Interval mapping uses two observable flanking markers to construct an interval within which to search for QTL. The optimum LOD thresholds were estimated by permutation of trait and marker data 1000 times with a walking speed of 2 cM. Composite-interval mapping (CIM) tests whether an interval between two markers contains a QTL while simultaneously controlling for the effect of proximal QTLs located outside the interval. CIM was performed using the standard model with a control marker number of *n*_*p *_= 5 and a window size of *w*_*s *_= 10 cM. We used 1000 permutation analysis to determine the optimum significance threshold of the LOD. Multiple-interval mapping (MIM) analyzes multiple marker intervals simultaneously to fit multiple putative QTLs. An initial MIM model was estimated by forward and backward marker selection with a probability of a partial *R*^2 ^set to 0.01. We then optimized QTL positions, searched for new QTLs, tested for existing QTL before saving the QTL model. The MIM model summary procedure estimated additive and dominance effects, the QTL likelihood ratio, the confidence interval around QTL positions, and partitioned the variance explained by QTL.

### Cloning of partial sequences of sodium channel (*kdr*) and acetylcholinesterase (*AChE*) genes in *An. funestus*

We cloned partial fragments of the two major target sites of insecticides used in malaria control, the acetylcholinesterase gene (*AChE*) which is the target site of carbamate and organophosphate insecticides and the voltage gated sodium channel gene (*kdr*) which is the target site of pyrethroids and DDT. Primers designed from a conserved region of the S6 region of the voltage-gated sodium channel gene AAK2 (5'-TGC GGA GAA TGG ATY GAA TC-3') and Ask2 (CTT AGC CTT GCT TTT GTC AAA) were used to amplify a 200 bp fragment of this gene from *An. funestus *genomic DNA. The PCR conditions were 96°C for 1 min followed by 30 cycles of 94°C for 30 s, 60°C for 30 s and 72°C for 30 s with a final 10 min extension at 72°C. The PCR product was ligated into pGEM-T easy vector and sequenced with M13 forward and reverse primers. From this 200 bp product, the Adf2 primer (5'-TGC AAA ATA GAG TCA TTG GTG AA-3') was designed and used with the Dip3 primer (5'-ATC ATC TTC ATC TTT GC-3') [36] to amplify a larger fragment of 1342 bp. This fragment was amplified and sequenced from individuals from the resistant FUMOZ-R strain and from field collected populations from Kenya, Mozambique and Malawi. A similar protocol was used to amplify a 1487 bp partial fragment of the acetylcholinesterase gene (*AChE*) in *An. funestus *using primers ANace-F1 (5'-CCG GGG GCG ACT ATG TGG AAC-3') and ANace-R3 (5'-GTT GCT GTT CGG GTT GTC CG-3'). The partial sodium channel and acetylcholinesterase of *An. funestus *sequences have been deposited in GenBank, accession numbers DQ534436 and DQ534435 respectively for *kdr *and *AChE*.

### Polytene chromosome *in situ *hybridization

A subset of 12 partial P450 Cytochrome P450 genes were chosen for physical mapping as increased activity of the P450 enzymes has been demonstrated in the FUMOZ-R resistant strain [17] and because this is an important pyrethroid resistance mechanism in other insects [27,37]. Half-gravid *An. funestus *females were collected from Kela village in Chikwawa district in southern Malawi. Ovaries were removed and preserved in Carnoy's fixative solution (3 ethanol: 1 glacial acetic acid by volume) and stored at -20° until use. The identity of specimens used in this study was confirmed by a rDNA diagnostic PCR which distinguishes four members of the *An. funestus *group [38]. Probes were prepared with the GIBCO BRL *in situ *hybridization and detection system, using the manufacturer's recommended protocol. Using fluorescence *in situ *hybridization (FISH), *An. funestus *clones were mapped on the five arms of the polytene chromosome. After hybridization, chromosomes were washed in 0.2× SSC and counterstained with YOYO-1 and mounted in DABCO antifade solution (Sigma) [19]. Fluorescent signals were detected using Zeiss LSM Pascal scanning confocal microscope.

## Authors' contributions

CSW (corresponding author) carried out the experiments, analyzed the data and wrote the manuscript. RH and MC provided all the samples for this study and contributed to the critical review of the manuscript. KS provided technical help, JM contributed to the gene cloning and the *in situ *hybridization, WCB contributed to the data analysis. JH is the PI of the program that funded the work and contributed to the critical review of the draft manuscript. HR contributed to the design of the study and critical review of the draft manuscript. All authors read and approved the final manuscript.

## Figures and Tables

**Table 1:** Markers with significant association with pyrethroid resistance for each family

**Significant loci**	**Chi-square**	**P-values**
*Family 1*		
7P6P4	5.92	0.015
*AChE*	5	0.03
*Family 2*		
7P6P4	6.02	0.013
*Family 3*		
7P6P4	6.32	0.011
*Family 4*		
7P6P4	7.33	0.019
3P6P3	5.9	0.032
AChE	5.5	0.035
AFND6	5.3	0.039
*Family 6*		
3BU82	5.87	0.03
6BU40	5.69	0.034
*Family 7*		
7P6P4	7	0.02
6BU40	5.8	0.034
*Family 10*		
No significant marker		
*Family 10X*		
7P6P4	6.5	0.01
3P6P3	6	0.022
*Family 11*		
7P6P4	9.78	0.004
3P6P3	7.46	0.015
AFND6	6.53	0.028
*Family 12*		
No significant marker		
*Family 16*		
7P6P4	7.45	0.01
3P6P3	6.9	0.029

**Table 2:** Multiple interval mapping estimates of QTL position and associated genetic, environmental, phenotypic, additive and dominance effects associated with pyrethroid resistance in *An. funestus*.

**σ_g_^2^(% σ_p_^2^)**	**σ_e_^2^(% σ_p_^2^)**	**σ_p_^2^**	**Nearest marker**	**Genetic distance (cM)**	**LOD**	**Effect**	**% σ_g_^2^**
**Family 4**							
14.72 (58.87%)	10.28 (41.13%)	25	7P6P4	0.1 (0.0–1.4)	1.09 0.18	A, -5.589 D, -0.660	31.1 -3.2
			G17	21 (20–22)	0.77 1.09	A, 2.1649 D, 3.6980	14.8 7.8
			ND10	75.2 (75.0–76.2)	0.27 0.95	A, 1.0312 D, 3.2745	1.4 7.1
**Family 11**							
15.86 (63.4%)	9.14 (36.6%)	25	7P6P4	7.3 (7.0–9.8)	2.26 0.02	A, -4.825 D, +2.762	49.8 13.6
**Family 6**							
2.428 (9.71%)	22.57 (90.29%)	25	3BU82	14.1 (14–16)	0.38 0.12	A, -2.4513 D, -0.7513	10.4 -0.8

σ_g_^2^, σ_p_^2^, σ_e_^2 ^respectively for genetic, phenotypic (in parentheses) and environmental variance; A for additive; D for dominance; confidence intervals for QTL position are in parentheses below the position estimate.

**Table 3:** Chromosomal locations of P450, Acetylcholinesterase (*AChE*) and knockdown resistance (*kdr*) genes in *An. funestus *and their orthologs in *An. gambiae*.

**Genes**	**Location in *An. funestus***	**Location in *An. gambiae***
CYP4G17	X:*	X: 5A–5C
CYP9K1	X*	X: 5A–5C
CYP6P2	2R: 9A–12C	2R: 11B–13E
CYP6P3	2R: 9A–12C	2R: 11B–13E
CYP6P4	2R: 9A–12C	2R: 11B–13E
CYP6P5	2R: 9A–12C	2R: 11B–13E
CYP6AA1	2R: 9A–12C	2R: 11B–13E
*CYP6M1*	*2R: 14A–15E*	*3R: 29A–30E*
*CYP6P1*	*2L: 27A–28E*	*2R: 11B–13E*
CYP6Z1	2L: 25A–27D	3R: 29A–30E
CYP6Z3	2L: 25A–27D	3R: 29A–30E
CYP9J12	3L*	3L: 43D–46D
CYP9J14	3L*	3L: 43D–46D
CYP4J10	3R*	2L: 22A–25D
AChE	2R: 9C–12C	2R:7C–10C
kdr	3R: 36A–37E	2L: 20C

* Exact division unknown since chromosomal arm defined only from the linkage map; Underlined and italized are the only genes where the synteny between *An. funestus *and *An. gambiae *was not preserved.

**Table 4:** Scoring of resistance phenotype in F2 progeny

**Family**	**No of progeny**	**No Dead**	**No. Alive**	**% mortality**	**No of survivors genotyped**	**No. of dead genotyped**
**♀Fang × Fumoz♂**						
Fam1	22	13	9	59.1	9	9
Fam3	24	13	11	54.2	11	11
Fam4	84	61	23	72.6	23	23
Fam6	82	51	31	62.2	31	31
Fam10	78	52	26	66.7	12	17
Fam11	131	70	61	53.4	33	33
**♀Fumoz × Fang♂**						
Fam2	27	15	12	55.6	12	12
Fam7	11	5	6	45.5	5	5
Fam10X	24	13	12	54.2	12	12
Fam12	15	8	7	53.3	7	7
Fam16	27	12	15	44.4	12	12

**Table 5:** Details on SNP markers scored by pyrosequencing method

**Markers**	**Forward primer 5'-3'**	**Reverse primer Biotine 5'-3'**	**Product size (bp)**	**Sequencing primer 5'-3'**	**Alleles**
**Chromosome X**					
BU21	TGATGGTGGATAAGCAGGTGTA	GAAGTTGCGCTTTACCGTGTC	77	CCGCAATCTGGCTAAT	A/T
G17	GATACGCTCGAGATGAAGTACCTT	CTGTACATCCTCGTTGATCTTGC	102	ATGAAGTACCTTGAGCG	A/G
4P9K1	GGCTAGCGGAACGAAATTGATAC	TCGTTCGCCACCATATCCAT	108	GAAAAGATAAGCTACGAAAC	A/G
**Chromosome 2**					
BU76	GGCACCGGTCAAGGATGTA	GCCAGGAGGTTACAAACCACAG	111	AGGATGTATCCAGCGTTA	A/G
7P6P4	GACTAAGCGATTCAATTGGAGGAT	CAACGGTAAGCATGGATACTCTCT	112	ATTCAATTGGAGGATATTT	A/G
3P6P3	GAGAATGACGGCCAGGTGA	GGGTACTTGCGAAGTGTTTCTGAA	142	CAATGTGATAAACGGTATG	C/T
*AChE*	TTGGTTCGTGCGGACATC	CGGTTCTGATTCTTTAAGCACAGC	175	AGTATTTTTGGGGACG	C/T
3BU82	GCACGGGAACTGGAAATGAT	GCATCGGAGCGTTTCCTATA	84	ACTGGAAATGATCGTTTA	A/G
6BU40	TTCCAGGCCAATGTCAGCA	AACGAAAACAAGGGGGAGAATG	78	CCAGGCCAATGTCAG	G/T
CYP6Z1	TGTCTGCACCAGCAGCATAGAAC	CGGGATTTCATCCAAATGCTA	121	CTTCCGCATTGTTTC	
BU29	GCACGGAGAAGGTGATCAACTA	GAGGTTGGTCGCAATTATTCG	188	CCTCCAGAAGAAGTACGAT	A/G
BU901	GGTGAGTTTTTTGGTTGCTTAATG	TCAATTTCTAAAATGGCATCAACA	97	GAGTTTTTTGGTTGCTT	A/G
1BU62	CGGTAAGTATAATATACGCCCAGT	GTTTGAGGAACGCATGAGC	75	TATAATATACGCCCAGTACA	C/T
**Chromosome 3**					
BU66	AAGTTCCAGGATGAAGGTGTGTT	GCCTTCGGGATGCCATTAT	129	ATGCGAGCTAGCCAA	A/G
BU92	ACGACTTCACCGGAGTCTCT	CGCTTCTTCCAGGGACTTATT	87	ACGACTTCACCGGAGTCT	C/T
5P9J12	CCAACAAATCAGTTCATCAGC	CTTTGGCGCGAAGGTAAA	97	TTGTAAAAGTGCTTAAAATG	A/C
2P9J14	TACCGTCTGTTTCCTTGGATAATG	ATTTCGGCGAAGAACAAATTTAG	80	GCTTGAAGTGGACCTG	G/T
BU021	TTTAGGGGTTCCAATTGACGC	ACCCATACCGGAACCAGTACCAC	56	ATTGACGCACTCGCT	G/T
1P9J10	TGCGTCTTTCGGTACAGGAT	AACGTATGTTCTTACCCACAACCA	141	AATCTTTACGGTTGTATCC	A/G
